# Comparative transcriptomics of the irradiated melon fly (*Zeugodacus cucurbitae*) reveal key developmental genes

**DOI:** 10.3389/fphys.2023.1112548

**Published:** 2023-01-17

**Authors:** Shakil Ahmad, Momana Jamil, Coline C. Jaworski, Yanping Luo

**Affiliations:** ^1^ School of Plant Protection, Hainan University, Haikou, Hainan, China; ^2^ Université Côte d’Azur, INRAE, CNRS, UMR ISA, Nice, France; ^3^ Beijing Academy of Agriculture and Forestry, Institute of Plant and Environment Protection, Beijing, China

**Keywords:** *Zeugodacus cucurbitae*, abiotic stress, insect physiology, gene expression, RNA interference (RNAi), developmental defects

## Abstract

Irradiation can be used as an insect pest management technique to reduce post-harvest yield losses. It causes major physiological changes, impairing insect development and leading to mortality. This technique is used to control the melon fly *Zeugodacus cucurbitae*, a major pest of Cucurbitaceae in Asia. Here, we applied irradiation to melon fly eggs, and the larvae emerged from irradiated eggs were used to conduct comparative transcriptomics and thereby identify key genes involved in the development and survival. We found 561 upregulated and 532 downregulated genes in irradiated flies compared to non-irradiated flies. We also observed abnormal small-body phenotypes in irradiated flies. By screening the 532 downregulated genes, we selected eight candidate genes putatively involved in development based in described functions in public databases and in the literature. We first established the expression profile of each candidate gene. Using RNA interference (RNAi), we individually knocked down each gene in third instar larvae and measured the effects on development. The knockdown of *ImpE2* ecdysone-inducible gene controlling life stage transitions–led to major body size reductions in both pupae and adults. The knockdown of the tyrosine-protein kinase-like tok (Tpk-tok) caused severe body damage to larvae, characterized by swollen and black body parts. Adults subject to knockdown of the eclosion hormone (*Eh_1*) failed to shed their old cuticle which remained attached to their bodies. However, no obvious developmental defects were observed following the knockdown of the heat shock protein 67B1-like (Hsp67), the insulin receptor (*Insr*), the serine/threonine-protein kinase Nek4 (Nek4), the tyrosine-protein kinase transmembrane receptor Ror (*Ror_1*) and the probable insulin-like peptide 1 (*Insp_1*). We argue that irradiation can be successfully used not only as a pest management technique but also for the screening of essential developmental genes in insects *via* comparative transcriptomics. Our results demonstrate that *ImpE2* and *Eh_1* are essential for the development of melon fly and could therefore be promising candidates for the development of RNAi-based pest control strategies.

## Graphical Abstract

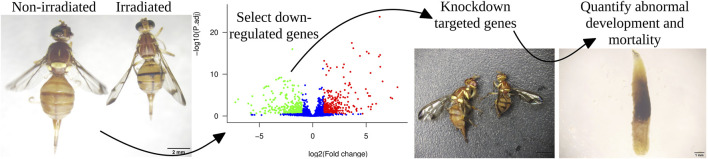

## 1 Introduction

Comparative transcriptomics are extremely useful to investigate gene functions even when genomic data are available as a source of bio information. High-throughput sequencing techniques have been widely used to investigate the function of genes linked with insect growth and development. A growing number of developmental transcriptomes have been published, including that of *Bactrocera dorsalis* (Shen et al., 2011; Chen et al., 2018), *Chrysomya megacephala* (Wang et al., 2015), *Trichopria drosophilae* (Zhou et al., 2019), *Cyrtotrachelus buqueti* (Yang et al., 2017), *Dendrolimus punctatus* (Yang et al., 2016), *Bombyx mori* (Ou et al., 2014) and *Phenacoccus solenopsis* (Arya et al., 2018).

The melon fly, *Z. cucurbitae* is a major insect pest of Cucurbitaceae plants worldwide, damaging vegetables and fruits of more than 130 species, including cucumber, pumpkin, watermelon, bitter gourd, tomato and eggplant (Khan et al., 2020). This invasive insect is widely distributed across climatic regions of Central and East Asia (including in China, Pakistan, Bangladesh, India, Nepal, Philippines, and Indonesia) and Oceania (including the Mariana Islands and New Guinea; Dhillon et al., 2005). It has been the target of multiple pest management programmes because of its high reproduction potential, adaptability, and invasion ability (Hoelmer and Daane 2020; Khan et al., 2020; González-Núñez et al., 2021). Insecticides, particularly organophosphates, have been used for decades as the main pest control strategy against the melon fly, despite being moderately effective and harmful to non-target organisms (Desneux et al., 2007).

Irradiation is a potential alternative pest management technique, not harmful to non-target insect species (Ayvaz and Yilmaz, 2015; Cai et al., 2018). Irradiation is used to limit fly growth and reproduction rather than causing severe mortality, because most fresh commodities could not sustain the enormous doses of radiation required to produce 100% acute mortality (Zhang et al., 2015). Nevertheless, insects can develop various physiological and morphological adaptive mechanisms protecting against irradiation (Armisén and Khila 2022). The physiological changes caused by irradiation may be due biochemical and molecular effects of irradiation. For instance, variations in gene and protein expression levels have been observed after irradiation (Marec et al., 2021). The heat shock protein is a possible irradiation marker since it responds to irradiation at high doses (Msaad et al., 2021). Irradiating pupae at a sterilizing dose may affect gene expression in adults (Chang et al., 2015). Such changes in expression levels may disturb pheromone signal processing and central energy generation in *Z. cucurbitae* pupae (Chang et al., 2015).

RNA interference (RNAi) is a reverse-genetic approach used to study insect functional genomics, recently used as a promising tool for gene knockdown in *Z. cucurbitae* (Ahmad et al., 2021b). For instance in *Z. cucurbitae*, the RNAi-mediated suppression of the *ZcVMP26Ab* gene expression resulted in increased desiccation and decreased hatch-ability of newly laid eggs (Li et al., 2021). Targeting key developmental genes *via* RNAi approaches could cause high levels of developmental defects and mortality in *Z. cucurbitae* (Ahmad et al., 2021b; Jamil et al., 2022). This could lead to potentially less costly and more efficient pest management programs which could be deployed at large scale while being specific and therefore not harmful to non-target insect species. Therefore, it is critical to understand the impacts of irradiation on *Z. cucurbitae* physiology and gene expression levels through a transcriptome analysis for the screening of potential target genes.

Throughout insect development, metamorphoses between life stages, *i.e*., larva to larva, larva to pupa and pupa to adult, are critical steps. These transitions are initiated and orchestrated by the 20-hydroxyecdysone (20E) (Žitňan et al., 2007), controlling events of apoptosis (type-I programmed-cell death–PCD) and autophagy (type-II PCD), as well as the remodeling of larval tissues and the differentiation of adult tissues from imaginal discs (Yin and Thummel 2005; Ryoo and Baehrecke 2010). The 20E-triggered transcriptional cascade is largely responsible for modulating the metabolic processes through autophagy caspase activity and cell dissociation during the larvae to pupae metamorphosis (Tian et al., 2010; Guo et al., 2012). Another critical step throughout insect development is ecdysis, the periodical shedding of the cuticle. This process is necessary for a marked increase in size, since sclerotized cuticles are virtually inextensible in insects. Cleaning the trachea is one of the most important physiological preparations to ecdysis (Kim et al., 2018).

Signaling pathways of insulin and insulin-like factors (IIS) play a significant role in insect body size regulation (Wu et al., 2016). The insulin receptor (InR) is a transmembrane receptor that activates signal transduction upon insulin binding (Sang et al., 2016). Growth inhibition and malformation were observed in ds*InR*-treated *B. mori* individuals (Zhang et al., 2014).

In the present study, we aim to identify genes impacted by irradiation in *Z. cucurbitae*, and responsible for major developmental defects, using comparative transcriptomics followed by targeted gene knockdown. We knocked down individually eight target genes putatively involved in insect growth, and we investigated the effects of knockdown on *Z. cucurbitae* development. We show that irradiation can successfully be used to identify candidate genes for the development of future RNAi-based pest management strategies.

## 2 Materials and methods

### 2.1 Insect rearing

*Z. cucurbitae* was reared in 35 cm × 35 cm × 35 cm cages in the insectary of Hainan University [Haikou, Hainan Province, China; temperature: 25°C ± 1°C; humidity: 60% ± 5%; photoperiod (L: D): 14:10 h] on a 1:3 yeast: sugar diet. Eggs were collected for the experiment by placing cucumber slices in Petri dishes in cages. A Petri dish containing pupae was placed in a new empty cage for adult emergence. Larvae were fed with artificial food following previous studies (Liu et al., 2020; Ahmad et al., 2021b).

### 2.2 ^60^Co radiation exposure

25 h old eggs were exposed to ^60^Co radiation at 50 Gy under free oxygen, at 1.0 Gy/min following previously published studies (Cai et al., 2018; Ahmad et al., 2021a). Experimental replicates were composed of three Petri dishes each containing 100 eggs (diameter of 100 mm; height 15 mm), and three replicates per treatment (irradiated *versus* control) were prepared. To increase hatching and prevent egg desiccation, Petri dishes were covered with a wet filter paper until hatching. Hatched larvae were immediately transferred to the same artificial diet as above. Abnormal phenotypes in irradiated samples were visually observed daily throughout the life cycle from 1st instar to adults. Twenty individuals of 3rd instar from each replicate of both irradiated and non-irradiated groups were frozen with liquid nitrogen and stored at −80°C before RNA extraction for transcriptomic analyses.

### 2.3 Transcriptome sequencing

1.5 µg of RNA was extracted from each sample. The NEBNext®UltraTM RNA Library Prep Kit for Illumina^®^ (NEB, United States) was used to prepare sequencing libraries. To attribute sequences to samples, unique index codes were added to each sample. Purification of mRNA from total RNA was performed with poly-T oligo-attached magnetic beads. Fragmentation was performed using divalent cations under elevated temperature in NEBNext First Strand Synthesis Reaction Buffer (5X). An M-MuLV reverse transcriptase (RNase H-) primer and random hexamer primer were used to produce first-strand cDNA. DNA polymerase I and RNase H were used to synthesize second-strand cDNA. The remaining overhangs were trimmed into blunt ends using exonuclease/polymerase. To prepare for hybridization, adenylation of the 3′end of DNA fragments was followed by ligation of the hairpin loop structure of NEBNext Adaptor. To select cDNA fragments with a similar length, the library fragments were purified using the AMPure XP system (Beckman Coulter, Beverly, United States) and 3 µl of USER Enzyme (NEB, United States) along with size-selected, adaptor-ligated cDNA at 37°C for 15 min followed by 5 min at 95°C (Utturkar et al., 2015). The PCR was carried out with Phusion High-Fidelity DNA polymerase, Universal DNA polymerase primers, and Index (X) primers. PCR products were purified using the AMPure XP system, and the Agilent Bioanalyzer 2100 was used to verify the quality of the library (Zhang et al., 2015).

We used the HiSeq 4000 PE Cluster Kit (Illumina) to cluster the index-coded samples on the Cluster Generation System following manufacturer instructions. The transcriptome was then sequenced using the Illumina Hiseq 4000 platform to generate 150 bp paired-end reads (Yang et al., 2021). Initial processing of raw fastq data (raw reads) was performed using custom Perl scripts (Ma et al., 2022). Low-quality reads and reads containing adapter and poly-N were first removed from the data. The Q30 and GC content were then calculated in the clean data. We used the clean, high-quality data for all downstream analyses. Reference transcriptomic data for *Z. cucurbitae* (GCF_000806345.2) was downloaded from the NCBI’s Sequence Read Archive database. A paired-end alignment of clean reads with the reference was performed using HISAT2 v2.1.0 (Jiang et al., 2022). The number of reads aligned to each reference gene (read depth) was counted using HTSeq v0.11.2. To measure gene expression level, we calculated the FPKM (number of Fragments Per Kilobase of transcript sequence per Millions base pairs sequenced) for each gene using the gene length and its read depth (Nawaz et al., 2018). RNA sequencing raw data has been deposited in the NCBI Sequence Read Archive (SRA; accession number GSE194002).

### 2.4 Differentially expressed genes (DEGs) and enrichment analysis

We used the DEGseq2 R package version 1.26.0 (Love et al., 2014) to test significant differences in gene expression levels between irradiated replicates and control replicates. The threshold for significantly different expressions was set at *p* < .05, a minimum of 1.5 fold change in expression level (|log2 (fold change)| ≥ .58), and an FPKM >1 in at least one of the six samples (Kumar et al., 2020). Gene expression levels were externally validated by qRT-PCR (Supplemenatry Figure S1). GO (Gene Ontology; http://www.geneontology.org/) significantly enriched pathways were identified using the R package ClusterProfiler with a *p*-value <.05 (Wu et al., 2021; Chen et al., 2021). KEGG (Kyoto Encyclopedia of Genes and Genomes) significantly enriched pathways were identified using the KEGG Automatic Annotation Server (KAAS, https://www.genome.jp/tools/kaas/) with *p* < .05 (Mao et al., 2005).

### 2.5 Expression profile and functional characterization of target genes

We observed major developmental defects in irradiated flies, including changes in body size in both pupal and adult flies. Therefore, we focused our mechanistic analysis of irradiation impacts on genes involved in developmental processes. We screened through the significantly downregulated genes in irradiated flies to identify genes previously associated with developmental pathways (GO and KEGG enrichment). Among the most downregulated genes, we selected eight candidates (*Insr*, *Hsp67*, *Tpk-tok*, *Nek4*, *ImpE2*, *Ror_1*, *Eh_1,* and *Insp_1*) for RNAi, based on their previously described role in developmental arrests of many insects species (Vlachou et al., 2006; Ragland et al., 2011; Mizoguchi and Okamoto 2013; Krüger et al., 2015; Lekha et al., 2015; Chang et al., 2019; Pérez-Lluch et al., 2020; Saraswathi et al., 2020).

To establish the temporal expression profile of these eight target genes, partial cDNA fragments of each gene were cloned. For this, total RNA was extracted using TRI reagent. The first strand of cDNA was synthesized using the HiScript R III 1st Strand cDNA Synthesis Kit. RNA was extracted from *Z. cucurbitae* flies at each developmental stage (eggs, 1st instar, 2nd instar, 3rd instar, pupae and adults). We analysed amplified products using agarose gel electrophoresis (1.2% agarose gel) and purified them using a Universal DNA Purification kit (Tiangen, China). We then performed RT-qPCR to verify the identity of gene fragments using SYBR R Premix Ex TaqTM II (TliRNaseH Plus) (Takara, Japan) on an ABI 7500 instrument (United States). Three biological replicates of each gene expression were performed using a PCR reaction containing 10 µl SYBER Green mix, 1 µl cDNA, 1 µl forward and reverse primers and 7 µl water (ddH2O). Gene expression levels were normalized using *EFα1* and *Actin* as internal controls (Jamil et al., 2022), and the 2^−ΔΔCT^ method was used to calculate the gene relative expression level (Livak and Schmittgen 2001). Similar protocols were used to evaluate gene silencing efficiency.

To assess the function of each target gene in development, we used RNAi to individually knock down each gene and measure the effects on body size, development, and survival of *Z. cucurbitae*. T7 RiboMAXTM Express RNAi System (Promega, United States) was used to synthesize dsRNAs specific to each gene. Each primer used for PCR contained a 5′ T7RNA polymerase binding site (GAA​TTA​ATA​CGA​CTC​ACT​ATA​GGG​AGA) followed by the gene-specific sequence. The obtained dsRNA products were purified following the manufacturer’s instructions, and the integrity and quantities of all synthesized dsRNAs were determined using 1.2% agarose gel electrophoresis. Purified dsRNAs were diluted in nuclease-free water, and their concentration was measured using the NanoDrop2000 spectrophotometer. The green fluorescent protein (GFP) dsRNA was used as a negative control. Eight groups of 3rd instar larvae of *Z. cucurbitae* were fed with an artificial diet treated with each gene-specific dsRNA for 24 h (h) and then shifted to another treated fresh food for another 24 h. The control group was fed with dsGFP-treated food (food treated with RNA silencing the Green Fluorescent Protein, used as a marker). The treated food was obtained by mixing 60 µl of dsRNA (1,000 ng/μl) with 6 g artificial of food. Three biological replicates were performed for each treatment (each of the eight genes) and control group, with 60 larvae in each replicate. Two larvae at 0 h, 12 h, 24 h, and 36 h post-feeding from each replicate were used for RNA extraction to quantify the silencing efficiency. Abnormal Phenotypes and survival ratios of the remaining 52 individuals were observed throughout the developmental stages until adult sexual maturity. All the primers along with gene id of the target genes are presented in Supplemenatry Table S2.

### 2.5 Statistical analysis

All statistical analyses were performed using R Core Team (2022). Independent linear regressions were performed to measure differences in the expression level of each target gene among life stages. The significance of the life stage fixed effect was tested using a one-way ANOVA, followed by mean comparisons using a Tukey test (function “TukeyHSD”, library “stats”; R Core Team, 2022). The effect of gene knockdown on the eight target gene expression levels at different times after feeding compared to control was tested using separate linear regressions for each gene. The treatment (knockdown vs. dsGFP control) in interaction with time after feeding (0, 12, 24 or 36 h) implemented as a factor were used as fixed effects. The significance of the interaction was tested with an ANOVA. This was followed by mean comparisons using the function “emmeans” (library “emmeans”; Lenth, 2022), testing differences in expression levels between control and knockdown treatment for each time.

## 3 Results

### 3.1 Quality of the transcriptomic assembly

The transcriptomic sequencing of *Z. cucurbitae* generated 19,084,528, 20,835,780 and 23,725,619 raw reads for the control replicates and 25,025,265, 26,719,132 and 25,411,483 raw reads the for irradiated replicates. After removing low-quality reads, 62,931,310 and 76,311,626 clean reads were generated in total for the control and the irradiated group respectively, and they were mapped to the reference genome. The percentage of total mapped genes ranged from 86% to 89% across replicates, reflecting the high sequencing quality (Table 1). The high-quality score (Q30) was over 92%, the GC content was above 43%, and the mapped ratio exceeded 87%.

**TABLE 1:** Transcriptomic sequencing and assembly quality.

Sample	Raw reads	Clean reads	Q30 (%)	GC content (%)	Total mapped	Mapped ratio (%)
Control1	19084528	18873766	92.16	42.44	16746692	88.73
Control2	20835780	20623263	92.72	42.88	17878306	86.69
Control3	23725619	23434281	92.37	43.23	20338612	86.79
Treatment1	25025265	24790216	92.36	43.46	21359250	86.16
Treatment2	26719132	26403833	92.77	43.58	22778586	86.27
Treatment3	25411483	25117577	92.63	43.23	22010532	87.63

### 3.2 Differentially expressed genes (DEGs)

1,093 genes showed a significant difference in expression levels between irradiated flies and control flies. Among these DEGs, 561 were upregulated and 532 were downregulated in the irradiated group compared to the non-irradiated group (Supplemenatry Figure S2). Gene expression levels were validated using qRT-PCR, and DEG expression levels were not significantly different in the transcriptomic data compared to qRT-PCR data (Supplemenatry Figure S1). Among all DEGs, two yolk protein vitellogenin-1 and vitellogenin-1-like were highly upregulated by ∼2,900 times and 600 times, respectively. Serine protease persephone-like, ctenidin-3-like, defensin-A-like, glycine-rich RNA-binding protein GRP2A-like and Gram-negative bacteria-binding protein 2-like genes were also amongst the most enriched DEGs in the irradiated group (by > 4,000, >700, >200, >600, and >150 times, respectively). Cell death abnormality protein 1, farnesol dehydrogenase-like, and 20-hydroxyecdysone protein genes were highly downregulated in the irradiated group, with expression levels 150, 65, and 54 times lower, respectively.

**FIGURE 1:**
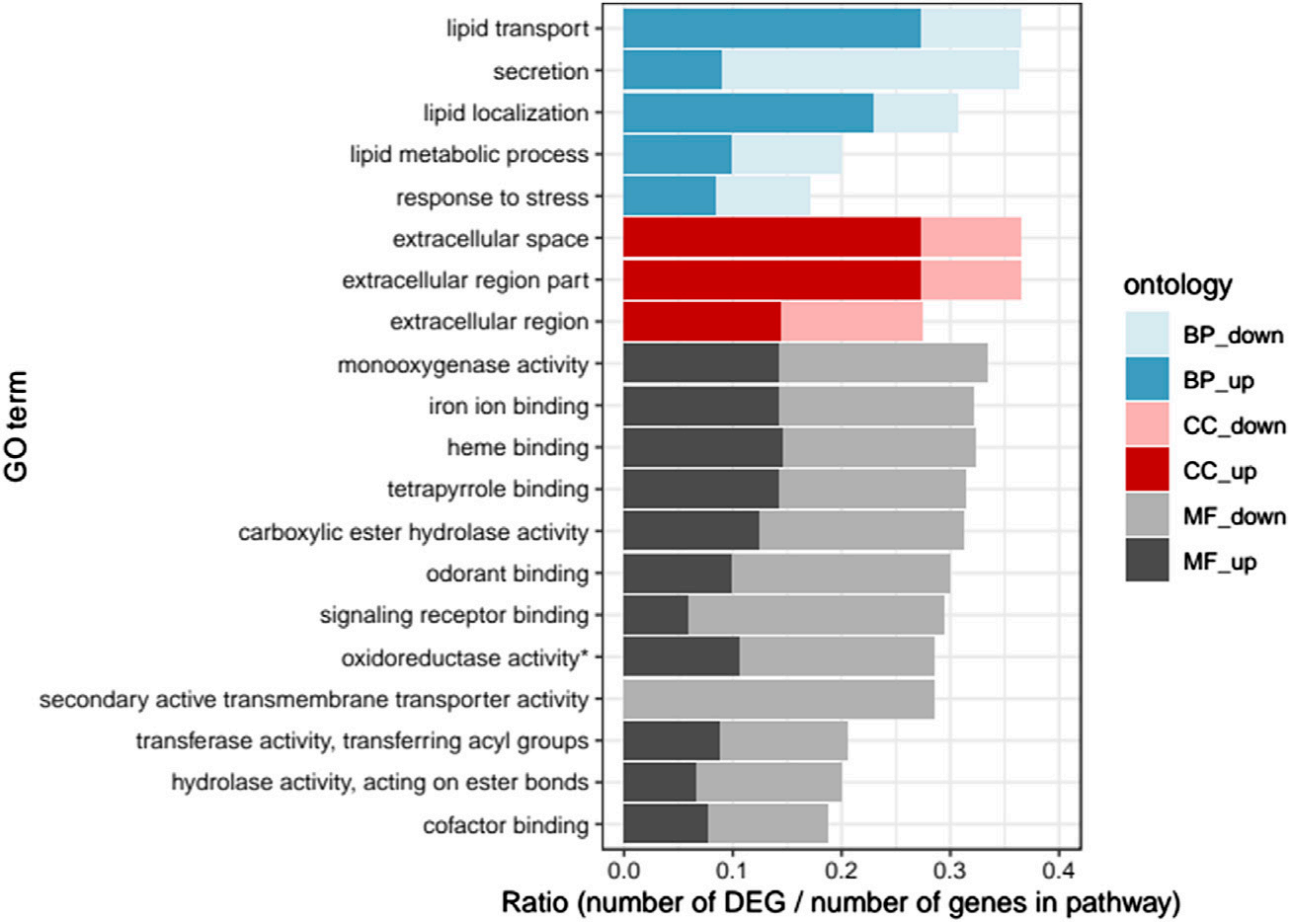
Gene ontology enrichment analysis: Ratio of the number of up- and downregulated differentially expressed genes (DEGs) over the total number of genes in each pathway, for the 20 significantly enriched GO pathways (*p* < .05). “BP”, biological process; “CC”, cellular component; “MF”, molecular function; “up", upregulated; “down”, downregulated (* oxidoreductase activity acting on paired donors, with incorporation or reduction of molecular oxygen).

Twenty GO terms were found significantly enriched in the DEGs (Figure 1). All pathways except one were enriched with both up- and downregulated DEGs. The most enriched pathways in biological processes were lipid transport and lipid regulation (mostly upregulated DEGs) as well as secretion (mostly downregulated DEGs). In cellular components, the most enriched pathways were extracellular space and extracellular region parts (mostly upregulated DEGs). Finally, in molecular functions, the most enriched pathways were monoxygenase activity and hydrolase activity as well as iron ion, heme, tetrapyrrole, odorant and signalling receptor binding functions, with slightly more down-than upregulated DEGs.

358 KEGG pathways were significantly enriched in irradiated flies, of which 164 were downregulated and 194 were upregulated. Among the 15% most enriched downregulated pathways, we found steroid biosynthesis, protein kinases, metabolism of xenobiotics by Cytochrome P450, exosome, and DNA replication proteins (Supplemenatry Figure S3B), which are pathways associated with developmental defects. Among the 15% most enriched upregulated pathways, we found apoptosis, AMPK signalling pathway, Cytochrome P450, enzymes with EC numbers, peptidases and inhibitors, and protein processing in endoplasmic reticulum (Supplemenatry Figure S3A), which are pathways associated with defence mechanisms.

### 3.3 Temporal expression of target genes

**FIGURE 2:**
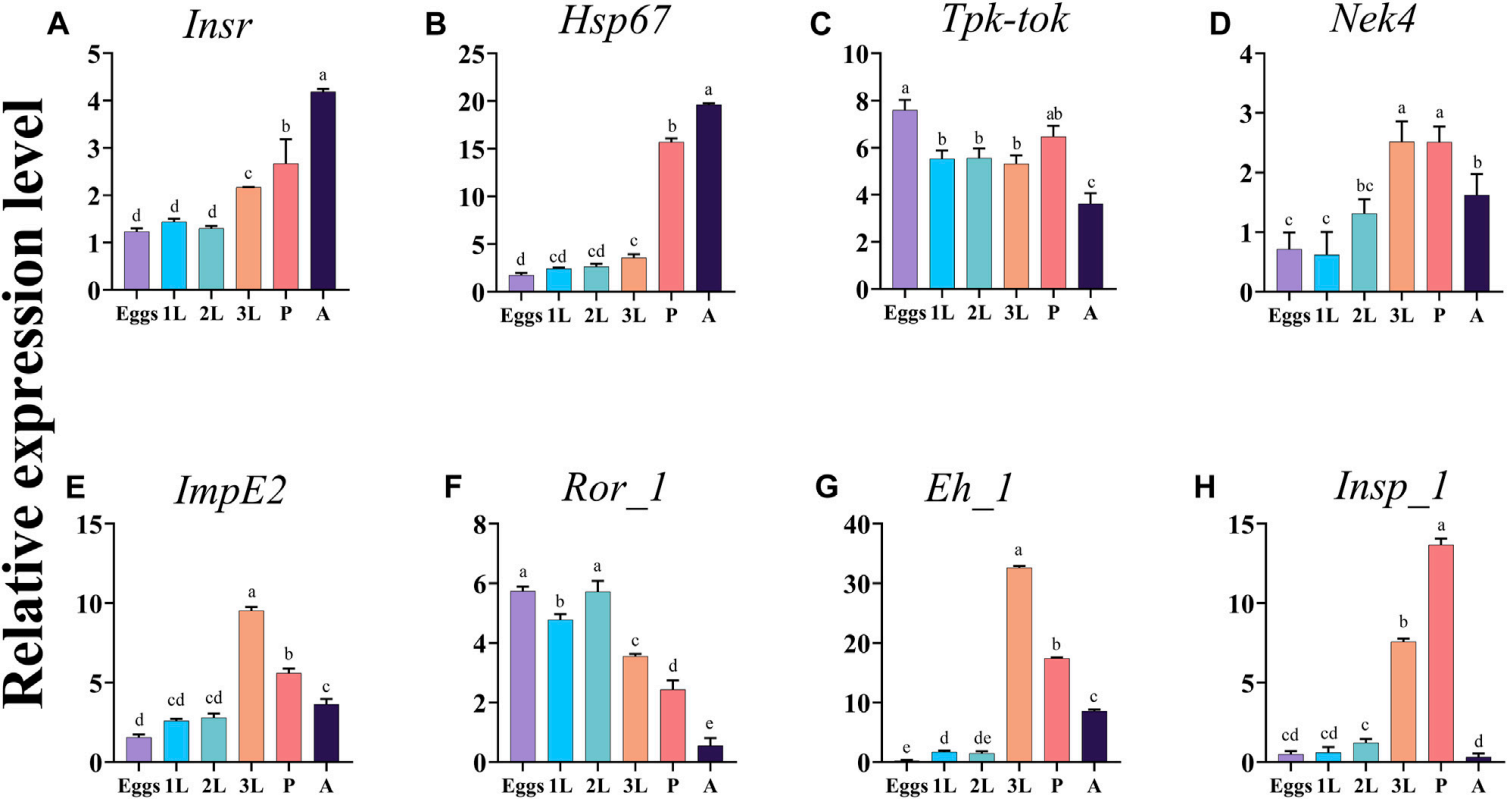
Expression profiles of the eight target genes *Insr*, *Hsp67*, *Tpk-tok*, *Nek4*, *ImpE2*, *Ror_1*, *Eh_1,* and *Insp_1* in different developmental stages of *Z. cucurbitae*. Eggs, 1st instar (1L), 2nd instar (2L), 3rd instar (3L), Pupae (P) and Adults (A). Values represent means ± SD. Different letters above the bars indicate significant differences at *p* < .05 (Tukey’s tests). *EFα1* and *Actin* were used as internal controls and the 2^−ΔΔCT^ method was used to calculate the gene relative expression level.

The expression analysis of the eight target genes *Insr*, *Hsp67*, *Tpk-tok*, *Nek4*, *ImpE2*, *Ror_1*, *Eh_1,* and *Insp_1* showed that their expression pattern varied among almost all developmental stages (Figure 2). *Insr* and *Hsp67* expression levels were the highest in adults, followed by pupae (Figures 2A, B). *Tpk-tok* was highly expressed in eggs, then its expression level remained the same in 1st, 2nd, and 3rd instar, then significantly increased in the pupal stage (Figure 2C). The expression levels of *Nek4, Eh_1* and *ImpE2* were highest in the 3rd instar, followed by pupae and adults (Figures 2D, E, G). *Ror_1* was expressed in all the developmental stages, and the highest expression was recorded in eggs and 2nd instar, followed by 1st, 3rd instar and pupae (Figure 2F). The expression level *Insp_1* was highest in 3rd instar followed by pupae and 2nd instar (Figure 2H).

### 3.4 Effects of dsRNA oral feeding on target genes expression

**FIGURE 3:**
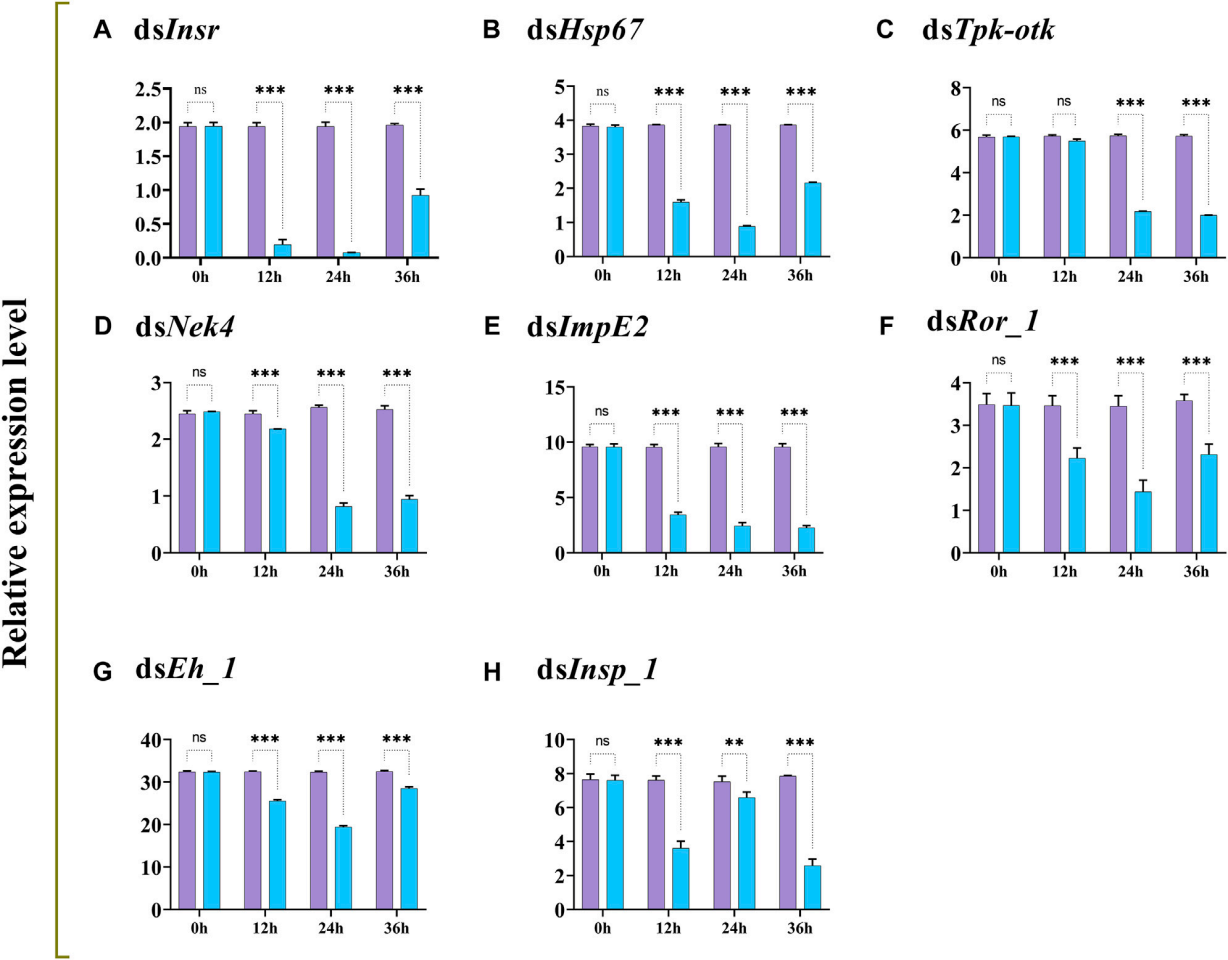
Relative expression profiles (mean ± SD; N = 3 replicates per treatment) of *Insr*, *Hsp67*, *Tpk-tok*, *Nek4*, *ImpE2*, *Ror_1*, *Eh_1* and *Insp_1* in *Z. cucurbitae* larvae at different times after feeding on gene-specific dsRNA (treatment; blue) compared to dsGFP (control; purple). *p < .05; **p < .01; ***p < .001; ns, no significant difference. *EFα1* and *Actin* were used as internal controls and the 2^−ΔΔCT^ method was used to calculate the gene relative expression level.

The transcript levels of *Insr*, *Hsp67*, *Tpk-tok*, *Nek4*, *ImpE2*, *Ror_1*, *Eh_1,* and *Insp_1* were significantly reduced 12–36 h after feeding on dsRNA-enriched food compared to the control group, except *Tpk-tok* which expression level was not significantly reduced before 24 h (Figure 3).

### 3.5 Genes silencing effects on insect development and survival

**FIGURE 4:**
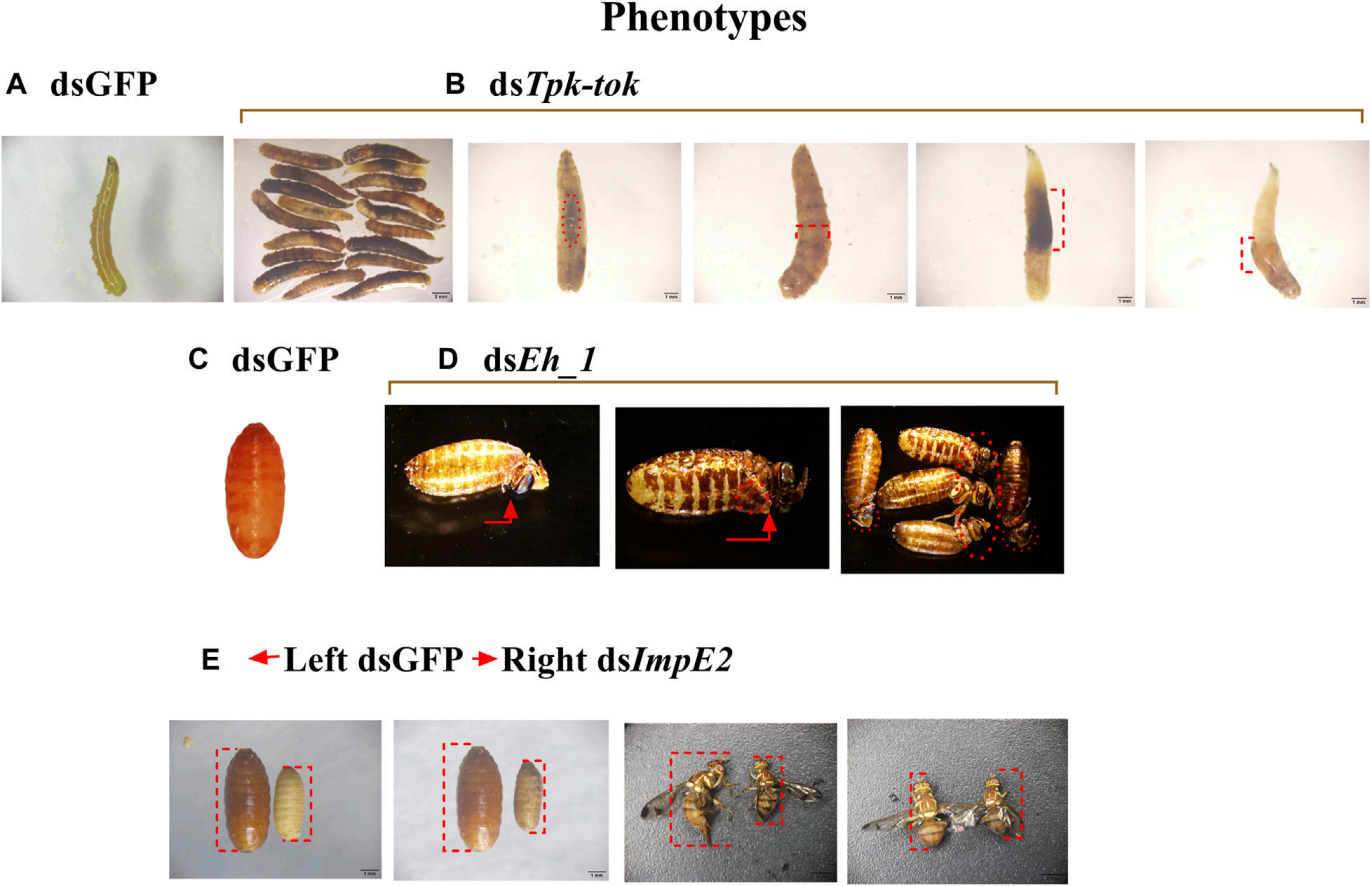
Developmental defects observed in *Z. cucurbitae* flies after the knockdown of **(B)** *Tpk-tok*, **(D)** *Eh_1* and **(E)** *ImpE2* genes. **(A)** 3rd instar of *Z. cucurbitae* after feeding on dsGFP-enriched food (control flies). **(C)** Normal pupae from the dsGFP control treatment. Red lines and circles represent the insect body’s damage, blockage, and swell portion in **(B)** and reduced body size in **(E)**, while red arrows show the old insect cuticle still attached to their body in **(D)**.

**FIGURE 5:**
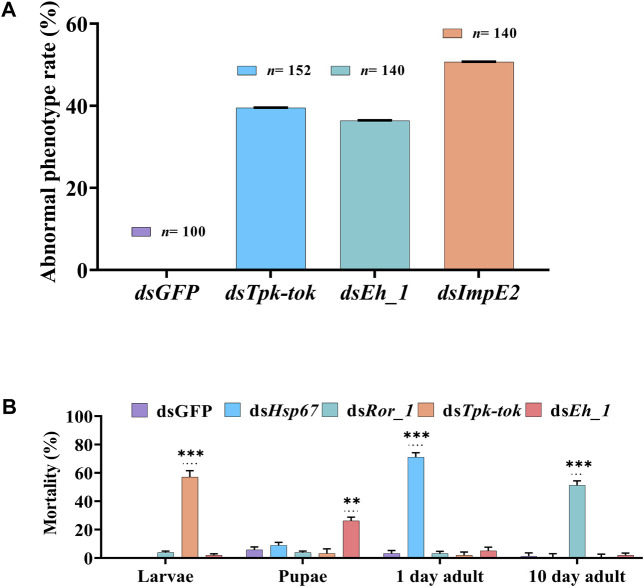
Abnormal phenotypes and Mortality observed in different developmental stages of *Z. cucurbitae*. **(A)** Percentage of observed abnormalities after dsRNA delivery of target genes and dsGFP (Control). **(B)** Percentage of insect mortality post feeding dsRNA of target genes compared to the control group. Values represent means % ± SD. Data were analysed using Duncan’s test.*p < .05; **p < .01; ***p < .001; ns no statistically significant difference compared to control.

Silencing of *Insr*, *Hsp6*, *Nek4*, *Ror_1,* and *Insp_1* did not visibly alter *Z. cucurbitae*’s phenotype at any developmental stage. However, the silencing of *Tpk-tok* caused severe defects in 39.5% of larvae 36 h after feeding compared to the control flies (Figures 4B, 5A). The larval body was distorted in the abdomen section. The middle part of the larvae near the anterior portion became blackish and 60% of the larvae died before pre-pupation stage (Figures 4B, 5A). Few larvae developed into the quiescent stage (about 96 h after ecdysis into final instar) when fed with *Tpk-tok* dsRNA. In contrast, *Eh_1-*silenced flies were able to molt to the pupal stage, and the shedding of the larval cuticle was completed. However, 26% flies fed with ds*Eh_1*-enriched food died at the pupal-adult eclosion stage, when the flies failed to shed their old cuticle and remain attached to their body, showing incomplete ecdysis (Figures 4D, 5A). Also 36.5% of the flies were recorded with the same abnormal phenotypes as when fed with ds*Tpk-tok* (Figures 4D, 5A). Finally, the knockdown of *ImpE2* had no effect on larval growth or survival but significantly reduced the body size in 50.7% of both pupal and adult flies compared to control flies (Figures 4E, 5A). A 12 h delay in adult eclosion was also observed due to *ImpE2* silencing compared to the control.

Knockdown of *Hsp67* decreased the survival of newly emerged adults by 71% compared to control-fed larvae (Figure 5). Similarly, the knockdown of *Ror_1* significantly decreased adult survival by 51% before the treated flies reached the sexual maturity stage. In contrast, the knockdown of *Tpk-tok* caused severe developmental defects in the larval stage compared to dsGFP leading to 57% of larval mortality. Similarly, the knockdown of *Eh_1* caused 26% mortality at the pupal stage (Figure 5).

## 4 Discussion

We identified and characterized the function of key genes involved in the development and survival of *Z. cucurbitae* using ^60^Co irradiation as a screening tool. We found 1,093 differentially expressed genes (DEGs) in irradiated flies, including 561 upregulated and 532 downregulated genes. Among these, we selected eight DEGs based on their putative function described in the literature (Vlachou et al., 2006; Ragland et al., 2011; Mizoguchi and Okamoto, 2013; Krüger et al., 2015; Lekha et al., 2015; Chang et al., 2019; Pérez-Lluch et al., 2020; Saraswathi et al., 2020), and we knocked down these genes individually to assess their role in the development of *Z. cucurbitae*. Successful knockdown of orally delivered dsRNA for target gene in artificial diet was reported previously in *Z. cucurbitae* (Ahmad et al., 2021b; Jamil et al., 2022). The knockdown of two genes, *ImpE2* and *Tpk-tok*, caused major developmental defects. This study reports key genes involved in the development of melon fly, a highly resistant insect pest and could be used for RNAi based management.

The expression level of the ecdysone receptor (EcR) target gene *ImpE2* was the highest in third instar larvae, followed by pupae and adults. *EcR* is essential for insect molting and metamorphosis (Yin and Thummel 2005; Žitňan et al., 2007; Ryoo and Baehrecke 2010). Due to tissue- and stage-specific mechanisms, *20E* is essential but sufficient for inducing autophagy and apoptosis in larval tissues, primarily through upregulation of several apoptosis and *Atg* genes (Lee et al., 2003; Yin and Thummel 2005; Liu et al., 2013). We observed that the suppression of *20E* through the knockdown of *ImpE2* at the larval stage caused precocious pupation and reduced pupal and adult body size. In insects, the larval stage determines the final body size analogously to the juvenile stage in humans (Edgar 2006; Mirth and Riddiford 2007). In general, insects with reduced body size suffer from either slow larval development or premature pupation, which shortens the larval developmental period (Colombani et al., 2003; Edgar 2006; Mirth and Riddiford 2007). Under *ImpE2* knockdown, we observed that larval body size was not affected but the transition to the pupal stage happened earlier than in control flies, which is a non-feeding stage of the insect. Thus, the smaller body size of flies is likely to be caused by precocious pupation due to the knockdown of *ImpE2.* Precocious or delayed pupation has been observed previously in many insects (Mirth et al., 2005; Layalle et al., 2008).

We found that suppressing *Tpk-tok* transcripts impaired larval development and ultimately caused mortality, highlighting its major role in *Z. cucurbitae* development and survival. Consistent to our findings, a previous study reported the involvement of tyrosine kinase (TK) in the development and survival of *Schistosoma mansoni* (Tavares et al., 2020). This pathway has an essential role in body development: receptor tyrosine kinase (RTK) signalling emerged as a key evolutionary strategy for transmitting extracellular information. Cells use this pathway to transduce extracellular cues, which contributes extensively to developmental processes. Previous studies reported that the ERK signalling pathway contributed to stopping the meiotic metaphase II in insects (Yamamoto et al., 2008), to egg fertilization (Yamamoto et al., 2013), and to vitellogenesis (Han et al., 2020). However, it is still unclear whether ERK signalling affects insect development or survival, as most of these studies investigated their role at the cellular level only. A knockdown of *Fer*, a TK from FES protein family in *Caenorhabditis elegans* (Nematoda: Rhabditidae), revealed that this protein is important for nematode epidermal differentiation (Putzke et al., 2005).

Similarly to *ImpE2*, the transcript level of *Eh_1* had the highest expression in third instar larvae, followed by pupae and adults and its silencing stopped the pupa to adult transition, when the flies failed to shed their old cuticle which remained attached to their body. We found that *Eh_1* transcripts were detected from egg to adult stage, and the mRNA levels were high in pupae and adults. Comparably, *EH* was found to be expressed in pre-pupae, five-day-old pupae, and early adults in *T. castaneum* (Arakane et al., 2008). The temporal expression indicates that *EH* likely exerts its function in the larvae-pupa-adult transition in *T. castaneum* (Arakane et al., 2008) and *L. decemlineata* (Shen et al., 2021), consistent with our findings in *Z. cucurbitae*. Also consistent with our results, the knockdown of *Eh_1* in the model insects *D. melanogaster* (McNabb et al., 1997; Clark et al., 2004; Krüger et al., 2015; Scott et al., 2020) and *T. castaneum* caused severe impairment in pupation and adult eclosion (Arakane et al., 2008). The ds*EH* delivery in *T. castaneum* through injection disrupted pre-ecdysis and consequently inhibited ecdysis during adult emergence but did not affect the larval-pupal transformation (Arakane et al., 2008). Altogether, these findings provide experimental evidence that the role of *Eh_1* in ecdysis during the pupae-to-adult transition seems conserved across insect species, including *Z. cucurbitae*.

Another gene of the insulin signalling pathway *Ror_1* was expressed in all the developmental stages. The highest expression was recorded in eggs and second instar larvae, followed by first and third instar larvae and pupae. When silencing *Ror_1* in larvae*,* no effects were observed on larval development, but most of the emerged adults died before reaching the sexual maturity stage. There were no differences in insect survival and development when *Nek4* and *Insp 1* were knockdown compared to non-treated larvae. Their expression was highest in the pupal stage, followed by third instar larvae. These results suggest different roles of each gene of the insulin-signalling pathway in *Z. cucurbitae*. The knockdown of *Insr* had no effects on the development and lifespan of *Z. cucurbitae*. Fontana et al. (2010) found no consensus on the function of *Insr* in insects, but Kenyon (2010) reported that the longevity of honeybee workers increased after silencing the IIS pathway, while it decreased following the suppression of *irs*. Knockdown of *HSP* caused up to 90% mortality in larvae and adults of the emerald ash borer *Agrilus planipennis* (Rodrigues et al., 2018). However, in the current study, the knockdown of *Hsp67* caused significant mortality in one-day-old adults but did not affect their physiology. A variety of proteotoxic tolerances is modulated by heat shock proteins (HSPs), originally identified as stress-responsive proteins (Feder and Hofmann 1999; Sørensen et al., 2003).

We have evidenced target genes involved in the development and survival of the major cucurbitae pest *Z. cucurbitae*. While enhancing the knowledge of the role of these genes in insect development, we suggest that *ImpE2,* involved in body regulation and *Tpk-tok* larval lethal gene could be potential targets for the development of RNAi-based pest control strategies against *Z. cucurbitae*, a major pest of a range of vegetables post-harvest. Such pest management strategies could be more efficient while less harmful to food crops and to non-target insects than chemical methods.

## Data Availability

The datasets presented in this study can be found in online repositories. The names of the repository/repositories and accession number(s) can be found in the article/Supplementary Material.
