# Supplementary material for: Comparative transcriptomics of the irradiated melon fly (Zeugodacus cucurbitae) reveal key developmental genes
Source: Front Physiol. 2023 Jan 17;14:1112548. doi: 10.3389/fphys.2023.1112548 (PMC9887199; doi:10.3389/fphys.2023.1112548)
Supplement: Supplementary file 1 [file DataSheet1.docx]

Supplementary Material

Comparative transcriptomics of the irradiated melon fly (*Zeugodacus cucurbitae*) reveal key developmental genes

**Shakil Ahmad, Momana Jamil, Coline C. Jaworski, Yanping Luo***

*** Correspondence:** Yanping Luo: [yanpluo2012@hainanu.edu.cn](mailto:yanpluo2012@hainanu.edu.cn)

*Abbrevations:*

DEG: differentially expressed gene

GO: gene ontology

KEGG: Kyoto Encyclopedia of Genes and Genomes

**1. Validation of DEGs expression levels through qRT-PCR**

We randomly selected 15 DEGs, including nine up- and six down-regulated. We constructed 15 pairs of primers for qRT-PCR analysis to validate the expression levels measured in transcriptome sequencing data. Table S1 lists these primers. The same RNA samples used for transcriptome sequencing analysis (section 2.3.) were used. Gene expression levels were normalized using *EFα1* and *Actin* as internal controls, and the 2^-ΔΔCT^ method was used to calculate the gene relative expression level.

**Table S1.** Description of primers used to validate gene expression level by qRT-PCR on 15 randomly selected DEGs.

| **#** | **Gene ID** | **Primer sequences** | **Tm** | **Product size** | **DEG fold change** | **DEG** |
| --- | --- | --- | --- | --- | --- | --- |
| 1 | LOC105216023 | TTCCTCATCTGCTTGGCTCT  GTACACAGCTGGAGCAGCAA | 60.10  60.21 | 180 | -5.814 | down |
| 2 | LOC114803523 | ACTTCGCTCTCGTTTTCGTC  TAGGGGTAAGCGGAGTAGGC | 59.62  60.59 | 202 | -3.212 | down |
| 3 | LOC105214460 | TCAAATTGTTCGCTGTCTGC  GCGATCGAGTGAGCATTGTA | 60.00  59.98 | 232 | -3.0171 | down |
| 4 | LOC105214441 | AACCCGTTCTGACCAAAGTG  CACAACTGGAGCAGTGGCTA | 60.01  60.05 | 155 | -2.4417 | down |
| 5 | LOC105211598 | ACTTCGCTCTCGTCTTCGTC  GCTGTATGGGTAAGCGGAGT | 59.75  59.22 | 206 | -1.6248 | down |
| 6 | LOC105214581 | ACCGACACTAATTGCGAACC  GCTGCTGTTGTTGTTCGTGT | 60.00  59.95 | 184 | -1.3571 | down |
| 7 | LOC105213841 | GGACGGAGATGCCGTATTTA  TGTATCAACGATGCCACGAT | 59.92  59.96 | 232 | 11.9914 | up |
| 8 | LOC105213847 | CCATAAAGGATGGTCGCAGT  TTTTCCGCAAATCTGGTTTC | 59.96  60.05 | 174 | 11.5391 | up |
| 9 | LOC105213509 | AATAAGCGATTCCGGGCTAT  CTGTTTTGACACCCACATGC | 59.92  60.01 | 152 | 6.26925 | up |
| 10 | LOC105215154 | GTTGCTGTTGGTGTGTTTGG  ACCTCCACCAAATGGAACAC | 60.05  59.68 | 214 | 9.62328 | up |
| 11 | LOC105213671 | AATTTCCACCAGCTGCTTTG  CCACATCATCTTTCGTGGTG | 60.25  59.96 | 183 | 3.65811 | up |
| 12 | LOC105213670 | TGAACGCTTCGTTTTGACTG  AAGCGGGTTGAACATAGTCG | 60.03  60.13 | 227 | 3.80228 | up |
| 13 | LOC105212138 | CACAGCTCAAAGCAAAGCAA  AAGGGATTCTGGCTCCAAGT | 60.32  60.07 | 167 | 5.05623 | up |
| 14 | Spbc4f6.14_1 | ATCGGATGGCGAGACTAATG  CGGGAAATAATCCCCCTTTA | 60.06  59.98 | 189 | 5.24786 | up |
| 15 | LOC105212139 | CGCTTTCTTCCTGTTCCAAG  TACAGTAACCGCCACGTTTG | 59.99  59.65 | 215 | 6.31382 | up |
| 16 | *EFα1* | CGTTGGTGTCAACAAGATGG TGCCTTCAGCATTACCTTCC |  |  |  | Control |
| 17 | *Actin* | GACTCGTACGTCGGTGAC  CTGTGTCATCTTCTCACGG |  |  |  | Control |
| 18 | GFP | CAGTGGAGAGGGTGAAG  TTGACGAGGGTGTCTC |  |  |  | Control |

Gene expression levels measured in the transcriptome data were not significantly different than those validated using qRT-PCR (Fig. S1) as tested with the 2^-ΔΔCT^ method.

**Table S2.** RNAi and qRT-PCR primers used for dsRNA preparation and expression pattern quantification of target genes.

| **Gene** | **Primer** | **Sequence** | **Size** |
| --- | --- | --- | --- |
| **LOC105220847**  **(*Hsp67*)** | dsRNA F  dsRNA R  qPCR-F  qPCR-R | GGATCCTAATACGACTCACTATAGCCACAAACTCATCGAGCTC  GGATCCTAATACGACTCACTATAGGTGCTGTTGCGTGCTATTG  GAGCCTGCAAATAGCGTAG  CTTCGACTGTTGCACTCAAC | 423  220 |
| **LOC105217849**  **(*Insr*)** | dsRNA F  dsRNA R  qPCR-F  qPCR-R | **GGATCCTAATACGACTCACTATAG**CGTTCATGGTCTACCCTCG  GGATCCTAATACGACTCACTATAGCATCGACACATTGACGTTCC  CGCCACACCCATTACAAC  CATGCCTTCCATCGTAGAG | 533  231 |
| **LOC105213376**  **(*Tpk-tok*)** | dsRNA F  dsRNA R  qPCR-F  qPCR-R | GGATCCTAATACGACTCACTATAGCACGCGCTGTTCTCATCAC  GGATCCTAATACGACTCACTATAGGCACTTCCTGTTTCCTGCTC  GCTTCACCTCTTCCAGTTGC  GCTCGTTCTCCTTGACTTG | 562  186 |
| **LOC105215510**  **(*Nek4*)** | dsRNA F  dsRNA R  qPCR-F  qPCR-R | GGATCCTAATACGACTCACTATAGCTAATGATAGCGCGCATGCGCAG  GGATCCTAATACGACTCACTATAGCAGTAATATGCGTTGTGGAGCC  GAGTCCCACCATGATCTCG  GATGGTGAACACGCGTTATC | 516  267 |
| **LOC105216289**  **(*ImpE2*)** | dsRNA F  dsRNA R  qPCR-F  qPCR-R | GGATCCTAATACGACTCACTATAGCAAGATGGTCAGCAGTTGGA  GGATCCTAATACGACTCACTATAGCACTTCTTCCGCTGTGTTCAG  CAGAATGCCGTTGCTGTAG  GTTCTGTTGGGTGGTGGTC | 456  165 |
| **ID: 105214701**  **(*Ror_1*)** | dsRNA F  dsRNA R  qPCR-F  qPCR-R | GGATCCTAATACGACTCACTATAGGGGTGTGGTGCTGAATAAG  GGATCCTAATACGACTCACTATAGCTGCTCATGCCAACATTCG  CAAAAGCTACCGTTGGAAG  GACATGTCTTCCCCGTACTC | 544  160 |
| **ID: 105218604**  **(*Eh_1*)** | dsRNA F  dsRNA R  qPCR-F  qPCR-R | GGATCCTAATACGACTCACTATAGATGTTCGCCACTACCAAATC  GGATCCTAATACGACTCACTATAGGCGATTGCGTTGAGAAAGG  TTGCCATCCATTGGACACTA  CCTTTGAATTTCAGGCAGGA | 299  155 |
| **LOC105212945**  **(*Insp_1*)** | dsRNA F  dsRNA R  qPCR-F  qPCR-R | GGATCCTAATACGACTCACTATAGGATGTCGATGAACCTGTTG  GGATCCTAATACGACTCACTATAGGTGTTGCAATAGGAGAGAATC  CGTTATGGCAGTTGTGTTGC  GAACGGAGAGTTCGCATAG | 366  190 |


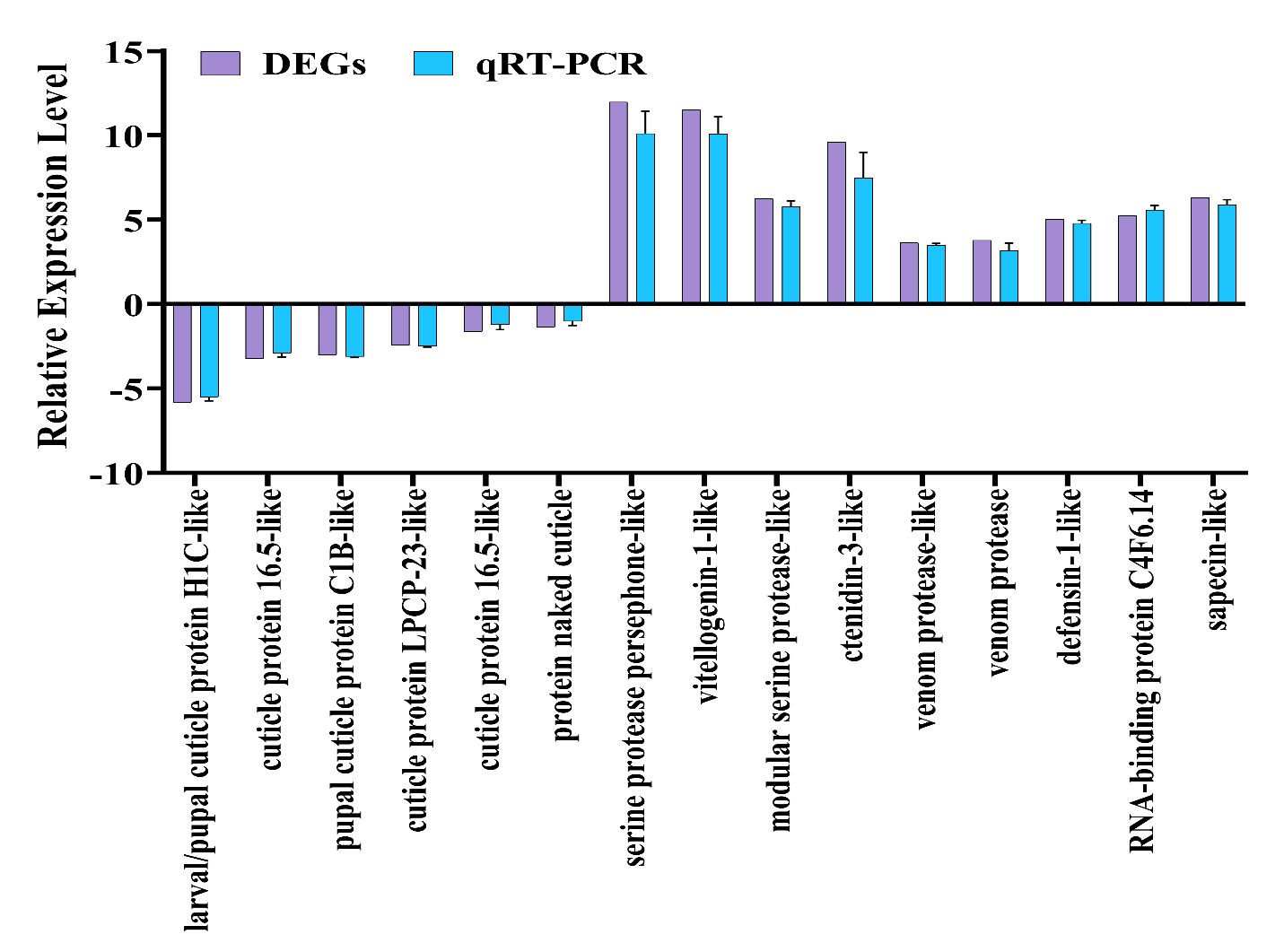


**Fig. S1.** Validation of 15 randomly-selected DEG expression levels using qRT-PCR data. Purple: gene expression levels calculated from RNA sequencing data; blue: gene expression levels calculated from qRT-PCR (mean % ± SD). The x-axis shows the 15 tested different genes. *EFα1* and *Actin* were used as internal controls and the 2^-ΔΔCT^ method was used to calculate the gene relative expression level.

**2. Supplementary figures**


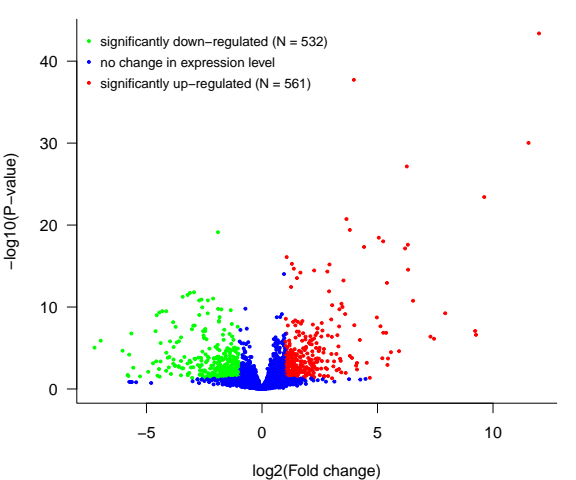


**Fig. S2.** Number of DEGs (*P*-value *<* 0.05 and |log2 (fold change in expression level)| ≥ 0.58) in irradiated *Zeugodacus cucurbitae* flies compared to non-irradiated control flies.


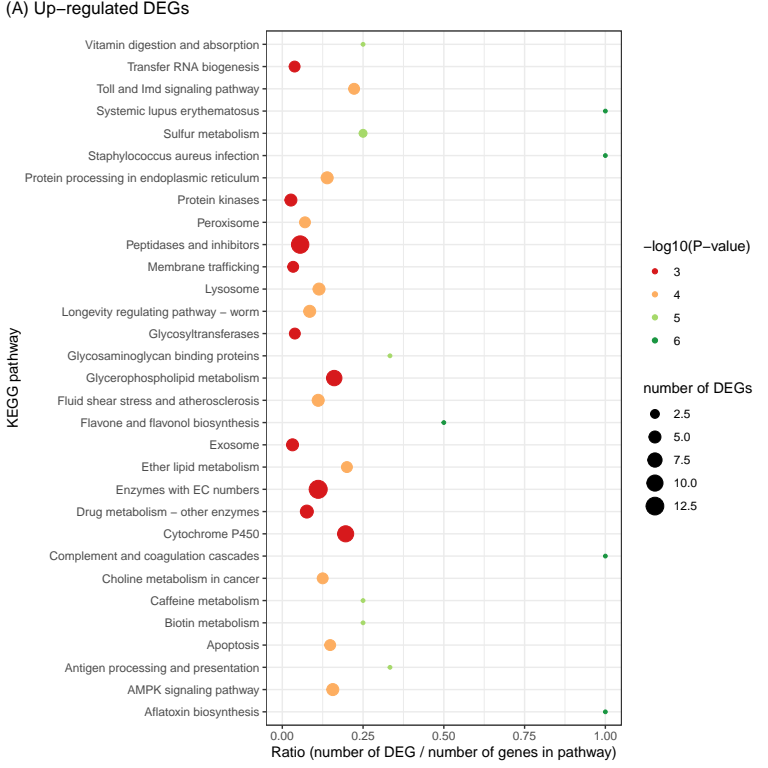

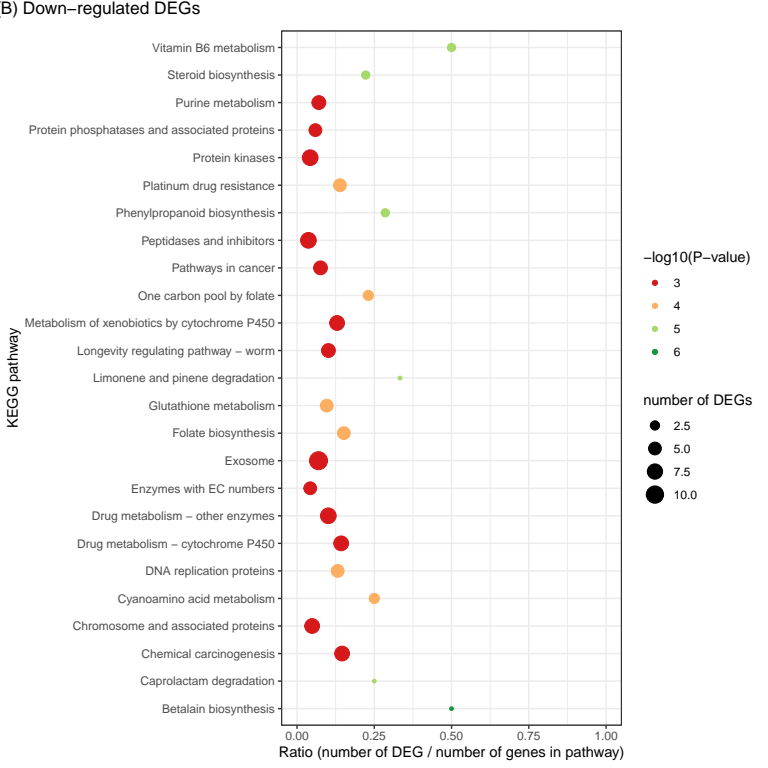
**Fig. S3.** KEGG enrichment analysis: Ratio of the number of (A) up-, and (B) down-regulated DEGs over the total number of genes in each KEGG pathway, for the 15 % most enriched significant (*P* < 0.05) KEGG pathways in irradiated *Zeugodacus cucurbitae* flies compared to non-irradiated control flies. The dot size shows the number of DEGs in each pathway, and the colour shows the degree of significance.
